# A systematic evaluation of payback of publicly funded health and health services research in Hong Kong

**DOI:** 10.1186/1472-6963-7-121

**Published:** 2007-07-30

**Authors:** Patrick Kwan, Janice Johnston, Anne YK Fung, Doris SY Chong, Richard A Collins, Su V Lo

**Affiliations:** 1 Research Office, Health, Welfare and Food Bureau, Government of the Hong Kong Special Administrative Region, Hong Kong, China; 2 Department of Medicine & Therapeutics, The Chinese University of Hong Kong, Prince of Wales Hospital, Hong Kong, China; 3 Department of Community Medicine, School of Public Health, The University of Hong Kong, Hong Kong, China

## Abstract

**Background:**

The Health and Health Services Research Fund (HHSRF) is dedicated to support research related to all aspects of health and health services in Hong Kong. We evaluated the fund's outcomes and explored factors associated with the translation of research findings to changes in health policy and provider behaviour.

**Methods:**

A locally suitable questionnaire was developed based on the "payback" evaluation framework and was sent to principal investigators of the completed research projects supported by the fund since 1993. Research "payback" in six outcome areas was surveyed, namely knowledge production, use of research in the research system, use of research project findings in health system policy/decision making, application of the research findings through changed behaviour, factors influencing the utilization of research, and health/health service/economic benefits.

**Results:**

Principal investigators of 178 of 205 (87%) completed research projects returned the questionnaire. Investigators reported research publications in 86.5% (mean = 5.4 publications per project), career advancement 34.3%, acquisition of higher qualifications 38.2%, use of results in policy making 35.4%, changed behaviour in light of findings 49.4%, evidence of health service benefit 42.1% and generated subsequent research in 44.9% of the projects. Payback outcomes were positively associated with the amount of funding awarded. Multivariate analysis found participation of investigators in policy committees and liaison with potential users were significantly associated with reported health service benefit (odds ratio [OR]_participation _= 2.86, 95% confidence interval [CI] 1.28–6.40; OR_liaison _= 2.03, 95% CI 1.05–3.91), policy and decision-making (OR_participation _= 10.53, 95% CI 4.13–26.81; OR_liaison _= 2.52, 95% CI 1.20–5.28), and change in behavior (OR_participation _= 3.67, 95% CI 1.53–8.81).

**Conclusion:**

The HHSRF has produced substantial outcomes and compared favourably with similar health research funds in other developed economies. Further studies are needed to better understand the factors and pathways associated with the translation of research findings into practice.

## Background

While research is believed to be essential in guiding improvements in health systems and developing new initiatives [1], there is growing recognition of the importance of measuring its returns [2-7]. In developed countries with long histories of publicly financing health research, providing evidence of benefit has increasingly become the criterion by which research agencies are evaluated and funding continuation assessed [8,9]. Evaluating research outcomes potentially allows the development of more effective strategies to increase the likelihood of the implementation of "successful" research [7].

Although the "best" way to evaluate research outcomes remains controversial, there is a general consensus that the evaluation should capture not only "academic" outputs (e.g. peer reviewed papers), but wider attributable health and socio-economic benefits that may include knowledge production, research targeting and capacity building, informing policy/decision making, behaviour change, product development, health and health service benefits and economic benefits [3-6,10]. Aiming to encompass these multiple dimensions, the "payback" framework, developed in the 1990s, is gaining popularity to become one of the most widely adopted models to evaluate health research funds internationally (Table 1) [11-17].

**Table 1:** Categories of outcome in the "payback" framework

1) Knowledge production: any accepted peer or non-peer reviewed publication (journal article, abstract, editorial, letter, book, book chapter, conference proceeding, report or others).
2) Use of research in the research system: a) the acquisition of formal qualifications by members of the research team, other research staff or prostgraduate students, b) career advancement for any members of the project team, and c) use of project findings for methodology in subsequent research by members of the project team
3) Use of research project findings in health system policy/decision making: project findings that could be used in policy/decision making at any level of the health service such as geographic level and organisation level
4) Application of the research findings through changed behaviour: changes in behaviour observed or expected through the application of findings to research-informed policies at a geographical, organisation and population level
5) Factors influencing the utilisation of research: estimated impact of research dissemination in terms of policy/decision making/behavioural change.
6) Health/health service/economic benefits arising from funded research: benefits that may or are expected to accrue from research funding such as improved service delivery; cost savings; improved health; or increased equity.

Although Hong Kong currently enjoys a relatively affluent economy, public funding for academic research was only formally established in the late 1980s. Funding allocation for projects in the fields of biology and medicine typically accounts for approximately one third of the total research budget [18]. Recognizing the need for locally relevant evidence to inform health policy and practice, the government has been supporting applied health research through the Health and Health Services Research Fund (HHSRF) since 1993. The fund aims to maximize population health, improve the quality of life, and enhance the standard and cost-effectiveness of the health system through funding research that generates new knowledge in areas of human health and health services [19].

To demonstrate accountability of public funding and to provide an evidence base for assessment of continuation of funding, we undertook for the first time a systematic evaluation of outcomes of research projects supported by the fund since it was established in 1993. The objectives of the study were to quantify the outcomes of completed research projects supported by the fund using the "payback" framework, in comparison with the more established health research funds abroad. We also explored factors associated with the impact of research outcomes on health policy and provider behaviour. Our results could have relevance for research funds planning similar evaluation exercises, particularly those that are less well established.

## Methods

### Research projects evaluated

**Figure 1:**
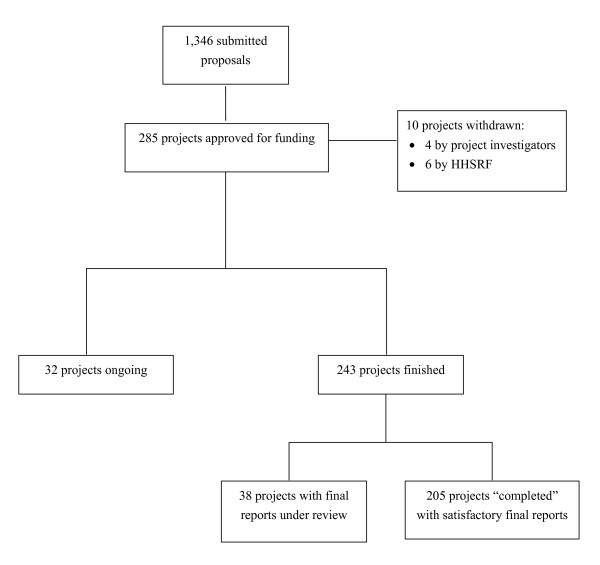
Flow diagram of projects funded by the Health and Health Services Research Fund (HHSRF) from 1993 to March 2006.

The HHSRF awards competitive grants to support a diverse range of health research projects, with an emphasis on public health and health services. Application to the fund is open to all professionals engaged in health research in Hong Kong, including those in academic institutions, public and private healthcare sectors. Applications are subject to a stringent peer review process by both international and local experts. Each finished project is required to submit a final report. When the final report is considered satisfactory after peer review, the project is considered "completed". As of March 2006, out of 1,346 applications received, a total of 285 projects worth HKD110.1 million (HKD 7.8 = USD 1) have been approved for funding. Of these, 205 projects worth HKD 73.1 million (HKD 7.8 = USD 1) have been completed and were the subject of this study (Figure 1). The mean funding amount per completed project was HKD356,585 (median HKD341,048, range HKD 6,110 to HKD 993,300). The usual funding ceiling per project was HKD 800,000 and the standard maximum duration was 24 months.

### Development of evaluation questionnaire

We adapted the payback evaluation framework questionnaire developed by the Health Economics Research Group at Brunel University, UK [14]. The revised questionnaire contained additional items drawn from literature review and consultation with local experts. A pilot test was conducted on 5 projects to assess the acceptability and feasibility of the questionnaire in the local setting. Minor modifications were made to better reflect the local context and readability. The final questionnaire [See Additional File 1] comprised six sections: a) knowledge production, b) use of research in the research system, c) use of research project findings in health system policy/decision making, d) application of the research findings through changed behaviour [12], e) factors influencing the utilization of research, and f) health/health service/economic benefits.

### Data collection

We sent the questionnaire in March 2006 to the principal investigators (PI) of the 205 completed projects (i.e. one questionnaire per project). To maximize return, at two to four week time intervals, we sent reminders to PIs via email, fax and/or by telephone. Where the PIs had left Hong Kong, co-investigators were invited to complete the questionnaire. The deadline for returning the completed questionnaires was end of June 2006. Returned questionnaires were checked for missing, inconsistent or unclear responses. Where necessary, PIs were contacted for further clarification.

### Data processing

We counted the total number of publications reported for each project by the PIs in the returned questionnaires and categorized publications into those published in peer reviewed or non-peer reviewed journals for analysis. For publications indexed by the Science Citation Index or Social Sciences Citation Index of the Institute of Scientific Information (ISI) [20,21], we retrieved the number of citations per publication as of July 2006, and the impact factor and rank within its subject category of the journal in which the publication appeared from the ISI website as of 2004. Based on the information provided by the PIs in the returned questionnaires, we counted the number of academic qualifications or career promotions acquired by members of the research team, the policies influenced, and behaviours changed or influenced for each project. Except for knowledge production, PIs were asked to estimate the contribution or expected impact of the research project on a categorical scale (considerable [≥ 75%], moderate [26–74%] or small [≤ 25%]). PIs were also asked to give evidence to support their impact assessment and participation in health-related policy/advisory committees. In addition, PIs were asked in the questionnaire whether there was any liaison with potential users of the research findings prior to or during the conduct of the project and the reply was counted as a dichotomous response.

We retrieved the investigator and project demographics, including type of project administering institution (university, hospital, or other agency), department or work place affiliation of the PIs, funding award in Hong Kong dollars and project duration, from the fund's electronic management database. Funding awards and project duration were categorized into tertiles respectively: low (HKD 6,110 – HKD 97,180), medium (HKD 98,940 – HKD 529,900), and high (HKD 532,242 – HKD 993,300); and short (4 – 15 months), intermediate (>15 – 24 months), and long (>24 – 56 months). To explore the impact of time on research publication we computed the duration from project completion in years dichotomizing the variable at the mean for the analysis.

### Statistical analysis

Univariate analyses by Chi-square test and Student t-test were used to test associations or differences between projects with and without returned questionnaires, and between the payback framework categories and funding award. We used logistic regression to identify factors associated with the uptake of research to inform policy decisions, leading to behavioural change or health service benefit adjusting for the number of peer reviewed publications, post-completion participation of the PI in heath-related policy/advisory committees, pre- or post-liaison with potential users, funding amount, project duration and type of administering institution. We used negative binomial regression, accounting for over dispersion in the data, to examine the association between the mean number of peer-reviewed publications and the fund award, project duration, years from project completion and the type of project administering institution.

We analyzed the data using SAS for Windows (version 9.1). P-values of <0.05 (two-sided) were considered statistically significant.

## Results

**Figure 2:**
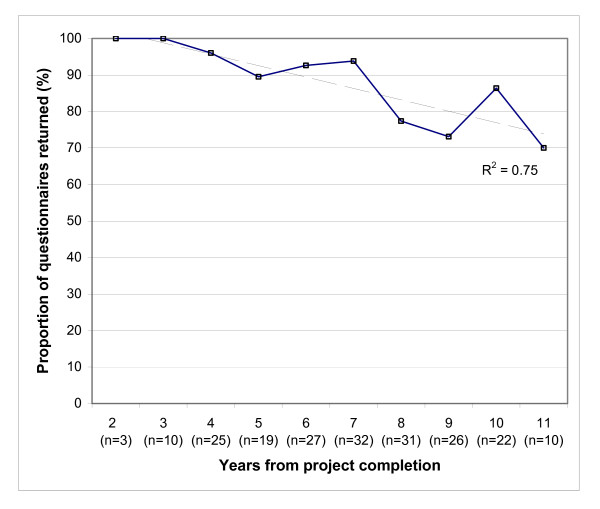
Questionnaire return rate by years from project completion.

Of the 205 questionnaires sent, 178 (86.8%) were completed and returned by investigators. The mean duration between project completion and questionnaire return was 6.34 years (median 6.25, range 1.75 to 10.92), which was significantly shorter than that for projects for which questionnaires were not returned (mean 7.76 years, median 7.75, range 3.25 to 10.83). The questionnaire response rate was inversely related to the time from project completion (p <0.001, R^2 ^= 0.75; Figure 2). In particular, significantly greater proportion of questionnaires were returned for projects completed 7 years or less compared with those completed longer than 7 years (94.0% [109/116] vs. 77.5% [69/89], odds ratio [OR] 4.51, 95% confidence interval [CI] 1.81–11.23).

Otherwise, there was no significant difference between projects for which questionnaires were returned and not returned in terms of the funding award, duration of project, or the type of administering institution (Table 2).

**Table 2:** Comparison of characteristics of projects with evaluation questionnaire returned or not returned

	Projects with questionnaires returned		Projects without questionnaires returned		
	N	%	N	%	P value
	
Administering institutions					
Universities	152	86.9%	23	13.1%	1.000
Hospitals and other agencies	26	86.7%	4	13.3%	
	
	Mean	SD	Mean	SD	p-value
	
Years from project completion	6.34	2.27	7.76	1.79	<0.001
Funding award (HKD)	355,163	275,492	367,338	275,381	0.832
Project duration (months)	20.6	10.1	20.1	8.6	0.770

### Research payback

#### Knowledge production

Among the 178 projects with returned questionnaires, 154 (86.5%) reported research publications. The mean number of publications per project was 5.4 (standard deviation [SD] 8.1). 70.8% (126 of 178) of projects reported peer reviewed publications with a mean number of 2.1 (SD 2.7) per project. Among the 377 peer-reviewed publications, 295 (78.2%) were published in journals that were listed in ISI Science or Social Sciences Citation Index. These publications had a mean of 1.9 (SD 4.0) citations per year (Table 3). 18.6% of the peer reviewed publications were published in one of the top three journals of their respective subject categories or in journals with impact factors greater than 7. The average expenditure per peer-reviewed publication was HKD 167,690.

**Table 3:** Association between funding award and research payback

		Funding Award*			
		
		Low	Moderate	High	P value
	N (%)		n(%)		
	
***Knowledge production***					
Projects with publications	154(86.5)	45 (79.0)	56 (87.5)	53(93.0)	0.09
	
	Mean(SD)		Mean (SD)		
	
Publications per project	5.4 (8.1)	2.8 (3.2)	5.0(5.6)	8.5(12.0)	<0.001
Peer reviewed publications per project	2.1(2.7)	1.1(1.7)	2.0(2.5)	3.3(3.3)	<0.001
Journal impact factor	3.0(3.9)	1.9(1.6)	2.7(2.7)	3.5(5.0)	<0.001
Journal ranking	19.7(24.7)	35.7(45.7)	17.0(16.7)	16.1(15.2)	0.02
Citations per year	1.9(4.0)	2.5(6.4)	1.3(1.4)	2.2(4.1)	0.23

	N (%)		n(%)		

***Research utilisation***					
Led to participation in health-related policy/advisory committees post research completion	34(19.1)	8(14.0)	13(20.3)	13(22.8)	0.47
Pre- and during- research process liaison with potential users	69(38.8)	19(33.3)	25(39.1)	25(43.9)	0.51
***Research targeting and capacity building***					
Generated subsequent research	80(44.9)	16(28.1)	29(45.3)	35(61.4)	0.002
Led to qualifications	68(38.2)	19(33.3)	20(31.3)	29(50.9)	0.06
Led to career advancement	61(34.3)	9(15.8)	25(39.1)	27(47.4)	0.001
***Informing policy and decision making***					
Findings used in policy making	63(35.4)	13(22.8)	23(35.9)	27(47.4)	0.02
Findings expected to be used in policy making	32(27.8)	5(11.4)	14(34.2)	13(43.3)	0.01
***Application of the findings through changed behaviour***					
Led to changes in behaviour	88(49.4)	23(40.4)	27(42.2)	38(66.7)	0.01
Expected to lead to changes in behaviour	36(40.0)	10(29.4)	16(43.2)	10(52.6)	0.22
***Health and health service benefit***					
Reported health service benefit	75(42.1)	23(40.4)	24(37.5)	28(49.1)	0.41
Expected future health service benefit	34(33.0)	8(23.5)	11(27.5)	15(51.7)	0.04

*Low = HKD 6,110–97,180; Moderate = HKD98,940–529,900; High = HKD532,242–993,300.

#### Research targeting and capacity building

Career advancement of research team members was reported for 34.3% (61 of 178) of projects (median 1 project team member per project, range 1 to 4); of these, the impact by the projects on this outcome was considerable (≥ 75%) in 13.2%. Acquisition of higher qualifications was reported for 38.2% of projects (68 of 178, median 1 postgraduate degree per project, range 1 to 6); of these, the impact by the projects was considerable in 57.3%. As an indication of research capacity building, 44.9% (80 of 178) of projects led to subsequent research (median 1 new research project per project, range 1 to 7); of these, the impact by the projects was considerable in 37.4%. In total, there were 115 new research projects worth HKD 123.0 million.

#### Informing policy, behaviour change, health service benefits

About one third (35.4%) of the projects reported impact on informing policy through treatment guidelines, treatment protocols, reference standards, and Cochrane reviews; many led to participation of PIs in health-related policy/advisory committees. An example was the inclusion of results from funded projects in formulation of guidelines on the use of non-steriodal anti-inflammatory drugs and COX-2 inhibitors by the 2005 US Task Force and the Maastricht-3 Consensus Report.^22 ^As another notable example, funding for the prevalence and economic impact of tobacco induced diseases, and effectiveness of smoking cessation has provided pivotal support for changes to tobacco legislation and regulation [23].

About one half (49.4%) of the projects were reported to have led to changes in behaviour or clinical practice in health service managers, providers and the general public. The PIs of 42.1% of the projects reported health service benefit from the funded projects including cost reduction through the adoption of cost effective treatment strategies, qualitative improvements in health service delivery, improve effectiveness of public health policies, and revenue gained from the selling of intellectual property rights. For instance, in addition to providing evidence to support legislation changes, results from the tobacco related studies have influenced the uptake of smoking cessation interventions by the public hospital authority and Department of Health in Hong Kong [23]. Univariate analyses revealed a significant funding dose response gradient (higher funding awards) with almost all aspects of the payback categories (Table 3).

#### Comparison with other research funds

**Figure 3:**
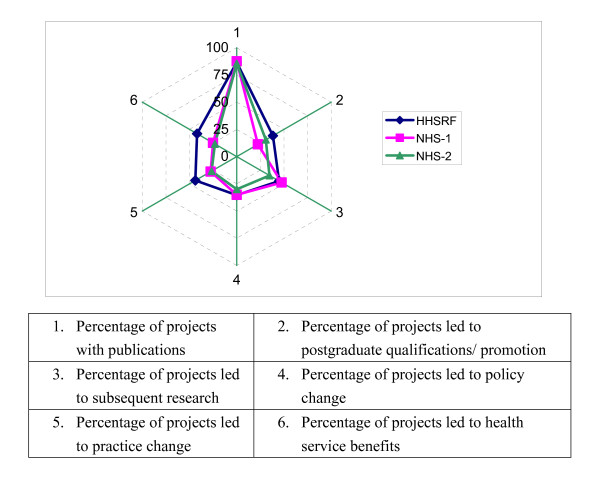
Comparison of the Health and Health   Services Research Fund (HHSRF) and two National Health Service Research and   Development Programmes (NHS-1 [12] and NHS-2 [13]) in various payback   categories.

Based on the modified payback framework we benchmarked the outputs and outcomes of HHSRF funded research against those reported by funds of other countries. Two National Health Service Research and Development Programmes in the UK [12,13] were selected for comparison. Although these evaluations focused on specific research programmes rather than an overall programme they were of similar funding scope to the HHSRF and also evaluated by the payback framework. There is a striking similarity between these three evaluations and the HHSRF compared favorably in all payback categories (Figure 3).

### Factors associated with the impact of research outcomes on health policy and provider behaviour

Table 4 shows that participation in health related policy/advisory committees post research completion and liaison with potential users pre- and during-the research process were independently predictive of reported health and health service benefit (OR_participation _= 2.86, 95% CI 1.28–6.40; OR_liaison _= 2.03, 95% CI 1.05–3.91), policy and decision-making (OR_participation _= 10.53, 95% CI 4.13–26.81; OR_liaison _= 2.52, 95% CI 1.20–5.28), and application of the findings through behaviour change (OR_participation _= 3.67, 95% CI 1.53–8.81). Increased funding award was also associated with increased behaviour change (OR_high _= 3.01, 95% CI 1.05–8.66).

**Table 4:** Factors associated with the uptake of research to inform policy decisions, lead to behavioural change and health service benefit

	Informing policy and decision making		Application of the findings through changed behaviour		Health and health service benefit	
	
	Adjusted Odds Ratio (95% CI)	P value	Adjusted Odds Ratio (95% CI)	P value	Adjusted Odds Ratio (95% CI)	P value
***Knowledge production***		0.79		0.88		0.08
No peer reviewed publications	1.00		1.00		1.00	
≤ 2.1 publications per project	0.80(0.32, 2.00)		1.22(0.54, 2.75)		2.38(1.05, 5.40)	
>2.1 publications per project	0.70(0.25, 1.96)		1.20(0.48, 2.98)		1.27(0.50, 3.21)	
***Research utilisation***						
Participation in health-related policy/advisory committees post research completion	10.53(4.13, 26.81)	<0.001	2.86(1.28, 6.40)	0.01	3.67(1.53, 8.81)	0.004
Pre- and during- research process liaison with potential users	2.52(1.20, 5.28)	0.01	2.03(1.05, 3.91)	0.03	1.09(0.56, 2.15)	0.79
Funding award*		0.17		0.56		0.04
Low	1.00		1.00		1.00	
Moderate	1.98(0.72, 5.39)		0.85(0.36, 1.99)		1.03(0.44, 2.42)	
High	3.18(0.95, 10.71)		1.34(0.47, 3.78)		3.01(1.05, 8.66)	
***Project duration (months)***		0.72		0.97		0.24
Short (4 – 15 months)	1.00		1.00		1.00	
Intermediate (15 – 24 months)	0.74(0.26, 2.12)		0.93(0.37, 2.31)		0.51(0.20, 1.29)	
Long (24 – 56 months)	1.04(0.34, 3.22)		1.01(0.37, 2.76)		0.90(0.33, 2.46)	
***Administering institution***		0.87		0.26		0.71
Universities	1.10(0.36, 3.33)		0.58(0.23, 1.48)		1.20(0.45, 3.18)	
Hospitals and other agencies	1.00	1.00	1.00			

*Low = HKD 6,110–97,180; Moderate = HKD98,940–529,900; High = HKD532,242–993,300

CI, confidence interval

Results from negative binomial regression analysis showed that significantly greater number of peer-reviewed papers published was found for projects with high (HKD 532,242 – HKD 993,300) funding awards (mean difference 0.76 compared with projects with low funding awards, 95% CI 0.19–1.33). Although projects with intermediate duration (15 – 24 months) appeared to have greater number of peer-reviewed publications compared to those with short duration (4 – 15 months), project duration was not significantly associated with such publications overall (Table 5).

**Table 5:** Factors associated with the publication of peer reviewed journal papers

	Mean Difference	(95% CI)	P value
***Funding award***			0.02
Low	0		
Moderate	0.32	(-0.19,0.83)	
High	0.76	(0.19,1.33)	
***Project duration (months)***			0.12
Short (4 – 15 months)	0		
Intermediate (15 – 24 months)	0.53	(0.01,1.04)	
Long (24 – 56 months)	0.49	(-0.05,1.04)	
***Years from project completion***			0.43
≤ 7 years	0		
> 7 years	0.16	(-0.23, 0.54)	
***Administering institution***			0.51
Universities	0.19	(-0.36, 0.73)	
Hospitals and other agencies	0		

*Low = HKD 6,110 – 97,180; Moderate = HKD98,940 – 529,900; High = HKD532,242 – 993,300.

CI, confidence interval

The effect of post research participation in health related policy/advisory committees and pre- or during-liaison with potential users is an essential component of the translation of research into practice, this effect is most likely strongest at the local level. Publication is necessary for international impact. The principal investigators reported numerous examples of research related impact ranging from the evaluation of unit based protocols and service delivery at the local departmental/hospital level; the development guidelines, programme planning and initiation applicable across Hong Kong; and inclusion of research outcomes in international treatment guidelines and protocols through the Cochrane library and WHO at the international level. Some examples are given in Table 6.

**Table 6:** Examples of impact of research outcomes on health policy and provider behaviour

*Local hospital/health service level*
≺ Improved reporting of unintentional child injury cases and liaison between the Hospital Authority Informatics and Accident and Emergency departments
≺ Improved liaison between the Education and Manpower Bureau and the Hospital Authority Child Psychiatry Services to address identification and treatment for children/adolescents at risk of suicide

*Hong Kong-wide level*
≺ Improved protocols and pathways for monitoring treatment progress and readiness for discharge from hospitals
≺ Development of a programme protocol for the Pneumoconiosis Compensation Fund Board
≺ Translation and validation of more than 17 internationally recognized questionnaires and scales into Chinese
≺ Provided scientific evidence to support the Hong Kong Tobacco Control Legislation

*International level*
≺ Research outcomes used in the support of Helicobacter pylori management guidelines (Maastricht-2 and Maastricht-3 Consensus Reports)
≺ Research outcomes guided Occupational Health Surveillance programmes in China and were influential in the inclusion of smoking as a health hazard for workers in China
≺ Inclusion in the Cochrane meta-analysis for the Cochrane Tobacco Addiction Review Group and Injury Prevention for Runners

## Discussion

Research is recognized as an essential feature of health care development and is increasingly used to influence all levels of health care provision. However, in most health care systems, including that in Hong Kong, the assessment of need, delivery of care and evaluation of preventive health and medical interventions has not been a strong feature. Health services research has the potential to substantially contribute to and influence health-related decision-making whether at the policy, practice, individual patient or population level. Well-structured research programmes are fundamental to adequate monitoring of the massive investments governments make in health care and to ensuring the appropriateness of future health care provision. Pressure to assess "value for money" in the use of public sector resources for research funding has risen in recent years [2-9]. Documenting the attributable research funding outputs or outcomes is essential for establishing the evidence base substantiating the "payback" or return on investment of public funds made available for health care research.

To our knowledge this is one of the largest studies of outcomes from a single public health research fund using the payback framework in terms of the number of individual projects evaluated. The robustness of findings of the present study has benefited from the high rate of return of evaluation questionnaires by the investigators (86.8%), reducing the risk of bias. Indeed among projects completed within the past 7 years, questionnaires were returned for nearly 95% of projects. The rate of questionnaire return dropped off significantly for projects completed more than 7 years before, which might reflect a lower incentive for investigators to respond due to the long period of time elapsed since project completion. This observation might have implication for the optimal timing to conduct studies of similar nature in future.

In spite of the fact that the total amount of funding awarded to the projects evaluated in this study is only a fraction of that awarded by more established research funds in other countries (for instance, the UK Medical Research Council's total research spending was GBP3.76 billion between 1995 and 2005) [24], our study documented a significant contribution of the HHSRF to knowledge generation through scientific publication at a level comparable to overseas research funds of a similar nature. This is demonstrated both in terms of the number of publications per funded project (HHSRF: mean 5.4 vs. Australian NHMRC [9]: mean 4.3) and 'value for money' in terms of expenditure per peer reviewed publication (HHSRF: mean HKD 167,690 vs. Australian NHMRC [9]: AUD 37,400 [approximately HKD 228,257]). However, it should be pointed out that these figures do not reflect the true total expenditure per publication, as the amount contributed by other funding sources, including those supporting the salaries of senior investigators, is unknown. Although a number of publications were in high impact journals we were unable to explicitly assess their individual impact on knowledge transfer of research into practice.

We found relatively fewer subsequent research projects (44.9%) generated by HHSRF-funded projects. This might reflect the relatively lack of funding opportunities for investigators in Hong Kong – it is estimated that only 0.69% of Hong Kong's gross domestic product (GDP) was spent on research and development in all disciplines of science and technology in 2004, while the corresponding figures were 2.78% in the UK, 2.67% in USA, 1.96% in Canada, and 1.69% in Australia [25]. Nonetheless, the HHSRF has been instrumental in supporting the development of research capacity and building a research culture in Hong Kong. In addition, HHSRF-funded projects have led to similar outcomes in terms of change in policy and self-perceived health service benefit compared with overseas funds evaluated by the same payback framework (Figure 3).

With the relatively large sample of projects evaluated, we were able to explore factors associated with research outcomes. It is perhaps not surprising to find that increased funding awards were significantly associated with greater outcomes in a range of payback categories. Perhaps more importantly, by multivariate analysis, we found that liaison with policy makers and integration of researchers in the policy formulation process, rather than publications or funding award, were the key factors influencing health behaviour change, health policy and health care benefit, confirming previous impressions [26,27].

The intrinsic limitations of the payback framework and the way it was applied should be recognized. Since information on project outcomes was provided by the investigators retrospectively, responses may reflect 1) recall bias, 2) over or under estimation of the effect of the research outcomes and 3) measurement error as the specific impact of the effect could not be explicitly measured and verified in all cases. There is inevitably a degree of subjectivity when researchers attribute their research findings to changes in health policy and behaviour, which involve a complex process with multiple factors at play, including political and economic consideration. It is also well recognized that researchers in different traditions or cultures vary in the way they conceptualize and explain the impact of their research [28]. It is likely that the heterogeneity in characteristics of the investigators sampled in this study has diluted the effect of the outcomes. In addition, respondent fatigue resulting from the length of the detailed questionnaire might also lead to response and information bias. In our study, the overall impact might also be underestimated, as PIs of projects completed more than 7 years prior to the survey were less likely to respond. Nonetheless, despite these shortcomings, with its growing acceptance by researchers and funding agencies, the payback framework represents a useful common tool by which the multiple dimensions of health research outcomes can be quantitatively and qualitatively measured, facilitating comparison in evaluation across different funds.

## Conclusion

In conclusion, despite the relatively young age and modest budget of the fund, the HHSRF has resulted in substantial outcomes as measured by a multi-level payback framework. As expenditure on research activities has to compete with alternative uses of scarce health services resources, the benefits arising from the investment should withstand rigorous evaluation. Quantifying the impact and payback of health services research and demonstrating the societal benefits is essential to providing the platform for continued policy support for health services research funding. Future research should focus on the overall long-term societal benefit factors that influence the uptake and translation of research into practice, and on improving the match between investigator initiated and policy directed research.

## Competing interests

The author(s) declare that they have no competing interests.

## Authors' contributions

PK and JJ designed and developed the study protocol, interpreted the results, and wrote the manuscript. AYKF designed and developed the protocol, interpreted the results, and participated in the revision of the manuscript. DSYC undertook the statistical analyses and participated in the revision of the manuscript. RAC developed the study protocol and participated in the revision of the manuscript. SVL conceived and supervised the study and helped to draft the manuscript. All authors read and approved the final manuscript.

## Pre-publication history

The pre-publication history for this paper can be accessed here:



## Supplementary Material

Additional file 1 Evaluation questionnaire. Questionnaire sent to principal investigators to evaluate research outcomes. Click here for file
