# Supplementary material for: A systematic evaluation of payback of publicly funded health and health services research in Hong Kong
Source: BMC Health Serv Res. 2007 Jul 30;7:121. doi: 10.1186/1472-6963-7-121 (PMC1952059; doi:10.1186/1472-6963-7-121)
Supplement: Additional file 1 — Evaluation questionnaire. Questionnaire sent to principal investigators to evaluate research outcomes. [file 1472-6963-7-121-S1.doc]

# Evaluation of

# Health and Health Services Research Fund (HHSRF)

# Project Demographics

# Please confirm if the following pre-completed information in this Section is correct. If not, please provide us with the correct information.

|  | Research project title: | | |  | | | |
| --- | --- | --- | --- | --- | --- | --- | --- |
|  |  | | | | | | |
|  | Research project reference no: | | |  | | | |
|  |  | | | | | | |
|  | Principal investigator: | | |  | | | |
|  |  | | | | | | |
|  | Affiliation of principal investigator: | | |  | | | |
|  |  | | | | | | |
|  | Administering institution: | | |  | | | |
|  |  | | | | | | |
|  | Project team members: | | |  | | | |
|  |  | | | | | | |
|  | Amount funded: | | |  | | | |
|  |  | | | | | | |
|  | Project duration: | Start date |  | | End date |  |  |

# If you have any questions about completing the questionnaire please contact Dr Richard Collins at 3150 8983, email: racollins@hwfb.gov.hk

# Thank you for your assistance.

# Please return by 10 April 2006 to the Research Office, Health Welfare and Food Bureau by email: rfs@hwfb.gov.hk

Note:

The focus of this evaluation is the various research funds administered by the Health, Welfare and Food Bureau and **NOT** the performance of individual researchers/project teams.

# /… A. PREVIOUSLY

# A. PREVIOUSLY NOTIFIED PUBLICATIONS

# Please check the pre-completed information in this Section about the publications from your research project, which are mostly those listed in your HCPF/HSRF research project final report. Please answer the questions about these publications and then complete the remaining Sections (B to H) using additional sheets if necessary.

**A1.** Please check the **previously notified publications listed** and:

1. delete any that were not, at least partially, a result of specific funding from the HCPF/HSRF;
2. please use one of the following letters to categorise each publication:

A = peer-reviewed journal article B = journal editorial C = journal letter

D = published abstract in journal E = book F = book chapter

G = non-peer-reviewed journal article H = published conference proceedings

I = publicly available full report J = others (please specify)

Please add a ‘**W**’ after the letter if it was a publication available **ONLY** on the web, for example, CW, EW.

1. for each publication cross (X) the appropriate box about HCPF/HSRF funding acknowledgement.

| Previously notified publications | **Category** | **HCPF/HSRF**  **Funding Acknowledged** | |
| --- | --- | --- | --- |
|  |  | **Yes** | **No** |
|  |  |  |  |
|  |  |  |  |
|  |  |  |  |
|  |  |  |  |
|  |  |  |  |
|  |  |  |  |
|  |  |  |  |
|  |  |  |  |
|  |  |  |  |

# /… B. PUBLICATIONS

# B. PUBLICATIONS NOT PREVIOUSLY LISTED

**B1.** Please list any **additional publications (not those listed in Section A)** that have resulted directly or indirectly from the research project.

Include any accepted publications that are in press but not any that are only at the submitted stage. For each publication please:

1. use one of the following letters to categorise it:

A = peer-reviewed journal article B = journal editorial C = journal letter

D = published abstract in journal E = book F = book chapter

G = non-peer-reviewed journal article H = published conference proceedings

I = publicly available full report J = others (please specify)

Please add a ‘**W**’ after the letter if it was a publication available **ONLY** on the web, for example, CW, EW.

1. for each publication cross (X) the appropriate box about HCPF/HSRF funding acknowledgement.

| Publications not previously listed | **Category** | **HCPF/HSRF**  **Funding Acknowledged** | |
| --- | --- | --- | --- |
|  |  | **Yes** | **No** |
|  |  |  |  |
|  |  |  |  |
|  |  |  |  |
|  |  |  |  |
|  |  |  |  |
|  |  |  |  |
|  |  |  |  |
|  |  |  |  |
|  |  |  |  |

# /… C. USE

# C. USE OF THE RESEARCH IN THE RESEARCH SYSTEM

**C1.** Has participation in the research led to additional formal qualifications for any members of the project team (as listed in page 1) or other research staff/postgraduate students etc., or is it likely to do so?

| Yes |  |  | No |  |
| --- | --- | --- | --- | --- |

If so, please give details.

| **I. Any members of the project team (as listed in page 1)** | | | | | |
| --- | --- | --- | --- | --- | --- |
| **Type of qualifications awarded to project team members** | **Number of qualifications** | | **Contribution from the research project**  **(please cross (X) the appropriate box)** | | |
|  | **Gained** | **Expected^*^** | **Considerable (≥75%)** | **Moderate**  **(26%-74%)** | **Small**  **(≤25%)** |
| For example:  MD | 1 |  | X |  |  |
| MD |  |  |  |  |  |
| PhD |  |  |  |  |  |
| MPhil |  |  |  |  |  |
| MSc/ MPH |  |  |  |  |  |
| Others (please specify) |  |  |  |  |  |
| **II. Any research staff, postgraduate students, etc.** | | | | | |
| **Type of qualifications awarded to research staff, postgraduate students, etc.** | **Number of qualifications** | | **Contribution from the research project**  **(please cross (X) the appropriate box)** | | |
|  | **Gained** | **Expected^*^** | **Considerable (≥75%)** | **Moderate**  **(26%-74%)** | **Small**  **(≤25%)** |
| MD |  |  |  |  |  |
| PhD |  |  |  |  |  |
| MPhil |  |  |  |  |  |
| MSc/ MPH |  |  |  |  |  |
| Others (please specify) |  |  |  |  |  |

^*^postgraduate degrees currently in progress

**C2.** Has participation in the research led to career advancement for **any members of the project team** **(as listed in page 1)**?

| Yes |  |  | No |  |
| --- | --- | --- | --- | --- |

| **Number of team members** | **Changes in post** | | **Contribution from the research project (please cross (X) the appropriate box)** | | |
| --- | --- | --- | --- | --- | --- |
|  | **From** | To | **Considerable (≥75%)** | **Moderate**  **(26%-74%)** | **Small**  **(≤25%)** |
| For example:  2 | Assistant Professor | Associate Professor |  | X |  |
|  |  |  |  |  |  |
|  |  |  |  |  |  |
|  |  |  |  |  |  |

# /… C3. Have

**C3.** Have the research project findings or methodology or theoretical developments generated subsequent research by **any members of the project team (as listed in page 1)?**

| Yes |  |  | No |  |
| --- | --- | --- | --- | --- |

If so, please give details of further grants, if any, and describe the contribution of your original research project to securing these funds.

| **Funder** | **Amount** | **The importance of the research project to securing later funding (please cross (X) the appropriate box)** | | |
| --- | --- | --- | --- | --- |
|  |  | **Considerable (≥75%)** | **Moderate**  **(26%-74%)** | **Small**  **(≤25%)** |
| For example:  Research Grants Council | $500,000 |  |  | X |
|  |  |  |  |  |
|  |  |  |  |  |
|  |  |  |  |  |
|  |  |  |  |  |

**C4.** Are you aware of any significant ways in which your research project has contributed to further research conducted **by others?** If yes, please indicate.

| Yes |  |  | No |  |  | Don’t know |  |
| --- | --- | --- | --- | --- | --- | --- | --- |

| **Project team** | **Research project title/topic** | **The importance of your research project to the further research (please cross (X) the appropriate box)** | | |
| --- | --- | --- | --- | --- |
|  |  | **Considerable (≥75%)** | **Moderate**  **(26%-74%)** | **Small**  **(≤25%)** |
|  |  |  |  |  |
|  |  |  |  |  |
|  |  |  |  |  |

**C5.** Please describe any contribution to further research that you have listed in **C3** or **C4** that is of particular importance.

|  |
| --- |

# /… D. USE

# D. USE OF RESEARCH PROJECT FINDINGS IN HEALTH SYSTEM POLICY/DECISION MAKING

**D1.** Research project findings can be used in policy/decision making at any level (please refer to examples listed in D3) of the health service.[[1]](#footnote-2)

Have the findings from your research project already been used in any such ways?

| Yes |  |  | No |  |  | Don’t know |  |
| --- | --- | --- | --- | --- | --- | --- | --- |

**D2.** Are there any reasons for expecting the findings to be used for future

policy/decision making?

| Yes |  |  | No |  |  | Don’t know |  |
| --- | --- | --- | --- | --- | --- | --- | --- |

**D3.** If you have replied **Yes** to either **D1 or D2** please give details of the use and/or expected use including: the **levels** at which policies/decisions were influenced; and the importance or impact of the research project’s findings to the adoption of the policy(ies)/decision(s).

Use the following letters to categorise the **geographical level(s)** and **organisational level(s)** influenced.

#### Geographical level

A = local (Hong Kong) B = national (China) C = international

#### Organisational level in local / national / international context

D = Legislative Council E = Health, Welfare and Food Bureau

F = Department of Health G = Hospital Authority

H = Hospitals/Clinics (please specify) I = Institutional departments (please specify)

J = Universities/schools (please specify) K = Professional organisations (please specify)

L = NGOs M = Others (please specify)

| Policy/Decision | **Geographical level** | **Organisational level** | **Degree of impact of research on policy/decision making**  **(please cross (X) the appropriate box)** | | |
| --- | --- | --- | --- | --- | --- |
|  |  |  | **Considerable (≥75%)** | **Moderate**  **(26%-74%)** | **Small**  **(≤25%)** |
| For example:  Smoke-free restaurants legislation | A | d,e,f | X |  |  |
| Triple therapy for H. pylori eradication | a,c | H (PWH), I (medicine), K (American College of Gastroenterology) |  | X |  |
|  |  |  |  |  |  |
|  |  |  |  |  |  |
|  |  |  |  |  |  |
|  |  |  |  |  |  |
|  |  |  |  |  |  |

# /… D4. Please

**D4.** Please give supporting evidence[[2]](#footnote-3)2 for claims that such health system policy(ies) or decision making(s) listed in **D3** may be attributed to in part by the research project findings.

| Policy/Decision | Evidence supporting change in policy(ies)/decision(s) |
| --- | --- |
|
| For example:  Smoke-free restaurants legislation | Legislative and other government documents |
| Triple therapy for H. pylori eradication | American College of Gastroenterology guidelines, Department of Medicine protocols |
|  |  |
|  |  |
|  |  |

**D5.** Have you participated in health-related policy/advisory committees as a direct outcome of this research? If yes, please give details.

| Yes |  |  | No |  |
| --- | --- | --- | --- | --- |

| **Name of health-related policy/advisory committees** | **Post title** | **Contribution from the research project** |
| --- | --- | --- |
| For example:  Advisory Council on Food and Environmental Hygiene | Member | Research project findings provide local information on food and environmental hygiene standards in local Chinese restaurants. |
|  |  |  |
|  |  |  |
|  |  |  |
|  |  |  |

# /… E. Application

# E. APPLICATION OF THE RESEARCH FINDINGS

# THROUGH CHANGED BEHAVIOUR

**E1.** Have the findings from your research project **already led** to changes, either directly or through the application of research-informed policies, in the behaviour of the following types of people^3^?

Please cross (X) the appropriate box.

| **Types of people** | **Yes** | **No** | **Don’t know** |
| --- | --- | --- | --- |
| Medical/allied health professionals/other providers |  |  |  |
| Health care managers |  |  |  |
| Health service users or the wider public |  |  |  |

**E2.** Do you **expect** the findings to influence practitioner or managerial behaviour or involvement of health service users or the public in the future?

Please cross (X) the appropriate box.

| **Types of people** | **Yes** | **No** | **Don’t know** |
| --- | --- | --- | --- |
| Medical/allied health professionals/other providers |  |  |  |
| Health care managers |  |  |  |
| Health service users or the wider public |  |  |  |

# /… E3. If

**E3.** If you replied **Yes** to either **E1** or **E2** please specify: the population whose behaviour has changed, the **level** at which any change occurred; how important the research project findings were in changing behaviour.

Use the following letters to categorise the **geographical level(s),** **organisational level(s),** and **population** in which behaviours have changed.

#### Geographical level

A = local (Hong Kong) B = national (China) C = international

#### Organisational level in local / national / international context

D = Legislative Council E = Health, Welfare and Food Bureau

F = Department of Health G = Hospital Authority

H = Hospitals/Clinics (please specify) I = Institutional departments (please specify)

J = Universities/schools (please specify) K = Professional organisations (please specify)

L = NGOs M = Others (please specify)

###### Types of population

N = Medical/ allied health professionals/ other providers O = Health care managers

P = Health service users/ the wider public

| Behaviour | **Geographical level** | **Organisational level** | **Types of population** | **Degree of impact of research on behavioural changes**  **(please cross (X) the appropriate box)** | | |
| --- | --- | --- | --- | --- | --- | --- |
|  |  |  |  | **Considerable (≥75%)** | **Moderate**  **(26%-74%)** | **Small**  **(≤25%)** |
| For example:  Triple therapy for H. pylori eradication | a | g | n | X |  |  |
| Increase in fruit consumption | a | j (HKU) | p |  | X |  |
|  |  |  |  |  |  |  |
|  |  |  |  |  |  |  |
|  |  |  |  |  |  |  |

/… E4. Please

**E4.** Please give supporting evidence for claims that such changes in behaviour listed in **E3** may be attributed to in part by the research project findings.

| Behaviour | Evidence supporting change in behaviour |
| --- | --- |
|
| For example:  Triple therapy for H. pylori eradication | The most widely prescribed H. pylori eradication regimen by HA doctors |
| Increase in fruit consumption | Survey results |
|  |  |
|  |  |
|  |  |

# /… F. FACTORS

# F. FACTORS INFLUENCING THE UTILISATION OF RESEARCH

# F1. Please state approximately how many conference/workshop presentations, press conferences/briefings, interviews (newspapers, magazines etc.) and/or other media presentations have been based on or resulted directly or indirectly from the findings of this research project.

| Dissemination activities | Number |
| --- | --- |
| Conferences/workshops primarily for academics |  |
| Conferences/workshops primarily for practitioners/service users |  |
| Press conferences/briefings |  |
| Interviews (newspapers, magazines) |  |
| Other media presentations (TV or radio) |  |
| Others (please specify) ____________________ |  |

# F2. Describe any of these presentations, or any other dissemination activities, that were particularly important in achieving utilisation of the research project’s findings.

| Dissemination activities | Brief description | Impact of these activities on policy/decision making/behavioural changes |
| --- | --- | --- |
|  |  |  |
|  |  |  |
|  |  |  |

# F3. Was liaison with potential users a factor in actual or future research utilisation?

#

# Liaison before starting

| Yes |  |  | No |  |
| --- | --- | --- | --- | --- |

# Liaison during the research project

| Yes |  |  | No |  |
| --- | --- | --- | --- | --- |

# F4. Please describe any aspects of such liaison and interaction that were particularly important.

|  |
| --- |

# F5. Describe any other factors (other than ones connected to the HCPF/HSRF) that account for the research being utilised, or for the lack of utilisation. These could include the timeliness or quality of the research project, the research project findings being taken up by the key stakeholders, etc.

|  |
| --- |

# /… G. HEALTH

# G. HEALTH/HEALTH SERVICE/ECONOMIC BENEFITS

# ARISING FROM FUNDED RESEARCH

# G1. Various possible health/health service/economic benefits from the application of research project findings can arise. These benefits include: improved service delivery; cost savings; improved health; an increase in values considered desirable e.g. equity.

#

# Have any such benefits already arisen as the result of the application of your research project findings?**^4^**

# Please cross (X) the appropriate box.

| Nature of health/health service/economic benefits | Yes | No | Don’t know |
| --- | --- | --- | --- |
| Cost reduction in the delivery of existing services |  |  |  |
| Qualitative improvements in the process of service delivery |  |  |  |
| Increased effectiveness of services, e.g. increased health |  |  |  |
| Equity, e.g. improved allocation of resources at a district/hospital level, better targeting and accessibility |  |  |  |
| Revenues gained from Intellectual Property Rights |  |  |  |
| Wider economic benefits from commercial exploitation of innovations arising from research and development |  |  |  |
| Economic benefits from a health workforce and reduction in working days lost |  |  |  |

# G2. Do you expect health/health service/economic benefits arising from the applications of your research project findings to be evident in the future?

# Please cross (X) the appropriate box.

| Nature of health/health service/economic benefits | Yes | No | Don’t know |
| --- | --- | --- | --- |
| Cost reduction in the delivery of existing services |  |  |  |
| Qualitative improvements in the process of service delivery |  |  |  |
| Increased effectiveness of services, e.g. increased health |  |  |  |
| Equity, e.g. improved allocation of resources at a district/hospital level, better targeting and accessibility |  |  |  |
| Revenues gained from Intellectual Property Rights |  |  |  |
| Wider economic benefits from commercial exploitation of innovations arising from research and development |  |  |  |
| Economic benefits from a health workforce and reduction in working days lost |  |  |  |

# G3. If you replied Yes to either G1 or G2 please specify, giving any supporting evidence: the nature of the benefits; how and why the benefits have accrued; and, where relevant, how many people have benefited.

| Nature of benefits | How and why benefits have accrued | No. of people benefited | Supporting evidence |
| --- | --- | --- | --- |
|  |  |  |  |
|  |  |  |  |

# /… H. COMMENTS

# H. COMMENTS

# H1. If you wish, please describe any other consequences of the research project not already covered. Include factors that have affected impact or, if appropriate, and where relevant, reasons for lack of journal publications.

|  |
| --- |

# H2. Please describe any other ways (not previously mentioned) in which funding for this research project has increased the value/impact of your research project (e.g. networking with researchers undertaking similar studies, greater attention being received from potential users etc.).

|  |
| --- |

# H3. Do you have any comments about the questionnaire itself?

|  |
| --- |

# H4. If this questionnaire has been completed by someone other than the principal investigator please give your name and contact details.

| **Name** | **Post title** | **Telephone number** | **Email address** |
| --- | --- | --- | --- |
|  |  |  |  |

# END

# Thank you for your help

1. Examples of the use of negative results from studies (despite adequate statistical power) should be included. [↑](#footnote-ref-2)
2. ^2^ Evidence of policy relevance could take many forms including: citing/using research project findings in policy documents, treatment guidelines and protocols, being members of a committee issuing a policy document or a treatment guideline, inclusion of findings in a contract or in a document from an audit, an inspectorial or an evaluative body etc. [↑](#footnote-ref-3)
